# Supplementary material for: Elevated Serum Levels of YKL-40, YKL-39, and SI-CLP in Patients with Treatment Failure to DMARDs in Patients with Rheumatoid Arthritis
Source: Biomedicines. 2024 Jun 25;12(7):1406. doi: 10.3390/biomedicines12071406 (PMC11274319; doi:10.3390/biomedicines12071406)
Supplement: Supplementary file 1 [file biomedicines-12-01406-s001.zip › biomedicines-3043876-supplementary/Supplementary Tables_V3.pdf]

**Table S1. Quantile regression for YKL-40**

| Variable | Quantile | betta | Lower confidence interval (95%) | Upper confidence interval (95%) |
|---|---|---|---|---|
| Intercept | 0.25 | -0.64856 | -1.46769 | 0.66251 |
| | 0.5 | -0.15329 | -1.10527 | 1.48076 |
| | 0.75 | -0.34173 | -1.68802 | 1.73685 |
| YKL-39 (ng/mL) | 0.25 | 0.32456 | -0.16672 | 1.03325 |
| | 0.5 | 0.6881 | 0.10197 | 0.85996 |
| | 0.75 | 0.70215 | 0.10541 | 1.51802 |
| SICLP (ng/mL) | 0.25 | 0.09502 | 0.03573 | 0.27412 |
| | 0.5 | 0.10731 | -0.02401 | 0.23954 |
| | 0.75 | 0.12045 | -0.00771 | 0.26558 |
| DAS28 score | 0.25 | 0.52213 | 0.12845 | 0.67822 |
| | 0.5 | 0.43258 | 0.19165 | 0.64631 |
| | 0.75 | 0.49231 | 0.31825 | 0.70834 |
| Age (yrs) | 0.25 | -0.00027 | -0.00128 | 0.01798 |
| | 0.5 | 0.00003 | -0.01987 | 0.01704 |
| | 0.75 | 0.01029 | -0.03105 | 0.02554 |
| Diseae Evolution (yrs) | 0.25 | 0.01116 | -0.02657 | 0.03185 |
| | 0.5 | -0.00502 | -0.04041 | 0.02311 |
| | 0.75 | 0.00487 | -0.02733 | 0.03957 |
| ANTI-CCP (UI/mL) | 0.25 | 0.00344 | 0.00074 | 0.00486 |
| | 0.5 | 0.00546 | 0.00208 | 0.00782 |
| | 0.75 | 0.00608 | 0.0037 | 0.00848 |
| RF, UI/mL | 0.25 | -0.00305 | -0.00523 | 0.0006 |
| | 0.5 | -0.00191 | -0.00537 | 0.00072 |
| | 0.75 | -0.00159 | -0.00631 | 0.00364 |

**Table S2. Quantile regression for YKL-39.**

| Variable | Quantile | betta | Lower confidence interval (95%) | Upper confidence interval (95%) |
|---|---|---|---|---|
| Intercept | 0.25 | -0.64856 | -1.46769 | 0.66251 |
| | 0.5 | -0.15329 | -1.10527 | 1.48076 |
| | 0.75 | -0.34173 | -1.68802 | 1.73685 |
| YKL-40 (ng/mL) | 0.25 | 0.32456 | -0.16672 | 1.03325 |
| | 0.5 | 0.6881 | 0.10197 | 0.85996 |
| | 0.75 | 0.70215 | 0.10541 | 1.51802 |
| SICLP (ng/mL) | 0.25 | 0.09502 | 0.03573 | 0.27412 |
| | 0.5 | 0.10731 | -0.02401 | 0.23954 |
| | 0.75 | 0.12045 | -0.00771 | 0.26558 |
| DAS28 score | 0.25 | 0.52213 | 0.12845 | 0.67822 |
| | 0.5 | 0.43258 | 0.19165 | 0.64631 |
| | 0.75 | 0.49231 | 0.31825 | 0.70834 |
| Age (yrs) | 0.25 | -0.00027 | -0.00128 | 0.01798 |
| | 0.5 | 0.00003 | -0.01987 | 0.01704 |
| | 0.75 | 0.01029 | -0.03105 | 0.02554 |
| Diseae Evolution (yrs) | 0.25 | 0.01116 | -0.02657 | 0.03185 |
| | 0.5 | -0.00502 | -0.04041 | 0.02311 |
| | 0.75 | 0.00487 | -0.02733 | 0.03957 |
| ANTI-CCP (UI/mL) | 0.25 | 0.00344 | 0.00074 | 0.00486 |
| | 0.5 | 0.00546 | 0.00208 | 0.00782 |
| | 0.75 | 0.00608 | 0.0037 | 0.00848 |
| RF, UI/mL | 0.25 | -0.00305 | -0.00523 | 0.0006 |
| | 0.5 | -0.00191 | -0.00537 | 0.00072 |
| | 0.75 | -0.00159 | -0.00631 | 0.00364 |

**Table S3. Quantile regression for SI-CLP**

| Variable | Quantile | betta | Lower confidence interval (95%) | Upper confidence interval (95%) |
|---|---|---|---|---|
| Intercept | 0.25 | 0.67631 | -0.30833 | 2.13099 |
| | 0.5 | 1.07283 | -0.44734 | 3.87012 |
| | 0.75 | 1.94206 | -0.4624 | 4.43181 |
| YKL-40 (ng/mL) | 0.25 | -0.02099 | -0.09611 | 0.24837 |
| | 0.5 | 0.28943 | -0.10289 | 0.50734 |
| | 0.75 | 0.31189 | 0.05813 | 0.455 |
| YKL-39 (ng/mL) | 0.25 | 0.01485 | -0.11565 | 0.37851 |
| | 0.5 | -0.12522 | -0.74423 | 1.13321 |
| | 0.75 | -0.23883 | -0.24662 | 0.79998 |
| DAS28 score | 0.25 | 0.55621 | 0.31005 | 0.73663 |
| | 0.5 | 0.46637 | 0.09979 | 0.9963 |
| | 0.75 | 0.57111 | 0.38092 | 0.99482 |
| Age (yrs) | 0.25 | -0.00109 | -0.03604 | 0.00492 |
| | 0.5 | -0.00572 | -0.05341 | 0.06078 |
| | 0.75 | 0.00085 | -0.03563 | 0.03731 |
| Diseae Evolution (yrs) | 0.25 | 0.01347 | -0.01604 | 0.05566 |
| | 0.5 | 0.0233 | -0.03371 | 0.06515 |
| | 0.75 | -0.02206 | -0.04813 | 0.03255 |
| ANTI-CCP (UI/mL) | 0.25 | 0.00093 | -0.00164 | 0.00349 |
| | 0.5 | 0.00011 | -0.00064 | 0.00377 |
| | 0.75 | 0.00794 | 0.0037 | 0.00884 |
| RF, UI/mL | 0.25 | -0.00696 | -0.00416 | 0.00117 |
| | 0.5 | 0.00196 | -0.00267 | 0.00655 |
| | 0.75 | 0.00425 | -0.00213 | 0.00875 |

**Table S4.** Factors associated with failure to treatment with DMARDs (Logistic Regression).

| Variables | Univariate | | | Multivariate | | |
|---|---|---|---|---|---|---|
| | Odds ratio | 95% CI | *p* | Odds ratio | 95% CI | *p* |
| Levels of the YKL-40 protein, ng/mL | 7.93 | 3.08 – 23.20 | <0.001 | 8.30 | 3.38 – 23.00 | <0.001 |
| Levels of the YKL-39 protein, ng/mL | 1.44 | 1.10 – 2.00 | 0.016 | 1.50 | 1.15 – 2.07 | 0.006 |
| Levels of the SI-CLP protein, ng/mL | 1.63 | 1.26 – 2.17 | <0.001 | 1.62 | 1.29 – 2.12 | <0.001 |
| Total doses of glucocorticoids, mg/day | 1.16 | 1.01 – 1.36 | 0.065 | 1.17 | 1.01 – 1.37 | 0.038 |
| ACPAs, IU/mL | 1.01 | 1.01 – 1.02 | 0.015 | 1.01 | 1.01 – 1.02 | 0.005 |
| HAQ-DI | 1.47 | 0.87 – 2.80 | 0.21 | Not relevant for the model | | |
| ESR (mm/h) | 1.03 | 0.99 – 1.07 | 0.19 | Not relevant for the model | | |
| Age, years | 0.96 | 0.92 – 1.01 | 0.14 | Not relevant for the model | | |
| Disease duration, years | 0.98 | 0.92 – 1.03 | 0.43 | Not relevant for the model | | |
| Use of Methotrexate | 0.32 | 0.03 – 1.97 | 0.28 | Not relevant for the model | | |
| Methotrexate weekly doses | 1.10 | 0.98 – 1.31 | 0.19 | Not relevant for the model | | |
| Use of Leflunomide | 1.56 | 0.48 – 5.18 | 0.46 | Not relevant for the model | | |
| Combined therapy | 0.57 | 0.18 – 1.75 | 0.34 | Not relevant for the model | | |

**Table S5.** Models 6 to 15 of the variables associated with treatment failure and CLPs and their comparison using DeLong test.

| Models | AUC | DeLong Test |
|---|---|---|
| Models of variables associated with treatment failure and chitinase-like proteins | | |
| Model 6: Variables associated with treatment failure and the YKL-40 and YKL-39 proteins. | 0.904 | <0.001 |
| Model 1: Variables associated with treatment failure excluding the 3 chitinase-like proteins. | 0.806 | |
| Model 7: Variables associated with treatment failure and the YKL-40 and SI-CLP proteins. | 0.907 | <0.001 |
| Model 1: Variables associated with treatment failure excluding the 3 chitinase-like proteins. | 0.806 | |
| Model 8: Variables associated with treatment failure and the YKL-39 and SI-CLP proteins. | 0.875 | 0.012 |
| Model 1: Variables associated with treatment failure excluding the 3 chitinase-like proteins. | 0.806 | |
| Models of chitinase-like proteins | | |
| Model 9: The 3 chitinase-like proteins. | 0.893 | 0.027 |
| Model 1: Variables associated with treatment failure excluding the 3 chitinase-like proteins. | 0.806 | |
| Model 10: YKL-40 protein. | 0.798 | 0.870 |
| Model 1: Variables associated with treatment failure excluding the 3 chitinase-like proteins. | 0.806 | |
| Model 11: YKL-30 protein. | 0.789 | 0.853 |
| Model 1: Variables associated with treatment failure excluding the 3 chitinase-like proteins. | 0.806 | |
| Model 12: SI-CLP protein. | 0.763 | 0.362 |
| Model 1: Variables associated with treatment failure excluding the 3 chitinase-like proteins. | 0.806 | |

| | | |
|---|---|---|
| Model 13: YKL-40 and YKL-39 proteins. | 0.864 | 0.166 |
| Model 1: Variables associated with treatment failure excluding the 3 chitinase-like proteins. | 0.806 | |
| Model 14: YKL-40 and SI-CLP proteins. | 0.860 | 0.205 |
| Model 1: Variables associated with treatment failure excluding the 3 chitinase-like proteins. | 0.806 | |
| Model 15: YKL-39 and SI-CLP proteins. | 0.841 | 0.403 |
| Model 1: Variables associated with treatment failure excluding the 3 chitinase-like proteins. | 0.806 | |

**Table S6.** Patients with RA included in the study groups (responders and non-responders) who tested positive and negative for CLPs (YKL-40, YKL-39, and SI-CLP) according to the cut-off point of the ROC Curves.

| YKL-40 | | YKL-39 | | SI-CLP | |
|---|---|---|---|---|---|
| Negatives<br>n (%)<br><br>Serum levels lower than 1.3808 ng/mL | Positives<br>n (%)<br><br>Serum levels greater than or equal to 1.3808 ng/mL | Negatives<br>n (%)<br><br>Serum levels lower than 3.2569 ng/mL | Positives<br>n (%)<br><br>Serum levels greater than or equal to 3.2569 ng/mL | Negatives<br>n (%)<br><br>Serum levels lower than 4.0627 ng/mL | Positives<br>n (%)<br><br>Serum levels greater than or equal to 4.0627 ng/mL |
| 86 (47.3%) | 96 (52.7%) | 105 (57.7%) | 77 (42.3%) | 114 (62.6%) | 68 (37.4%) |
| Non-responders RA patients who tested positive for all 3 chitinases: 33 (35.86%) | | | | | |
| Responders RA patients who tested negative for all 3 chitinases: 51 (56.67%) | | | | | |
