# Supplementary figures and images for: Elevated Serum Levels of YKL-40, YKL-39, and SI-CLP in Patients with Treatment Failure to DMARDs in Patients with Rheumatoid Arthritis
Source: Biomedicines. 2024 Jun 25;12(7):1406. doi: 10.3390/biomedicines12071406 (PMC11274319; doi:10.3390/biomedicines12071406)

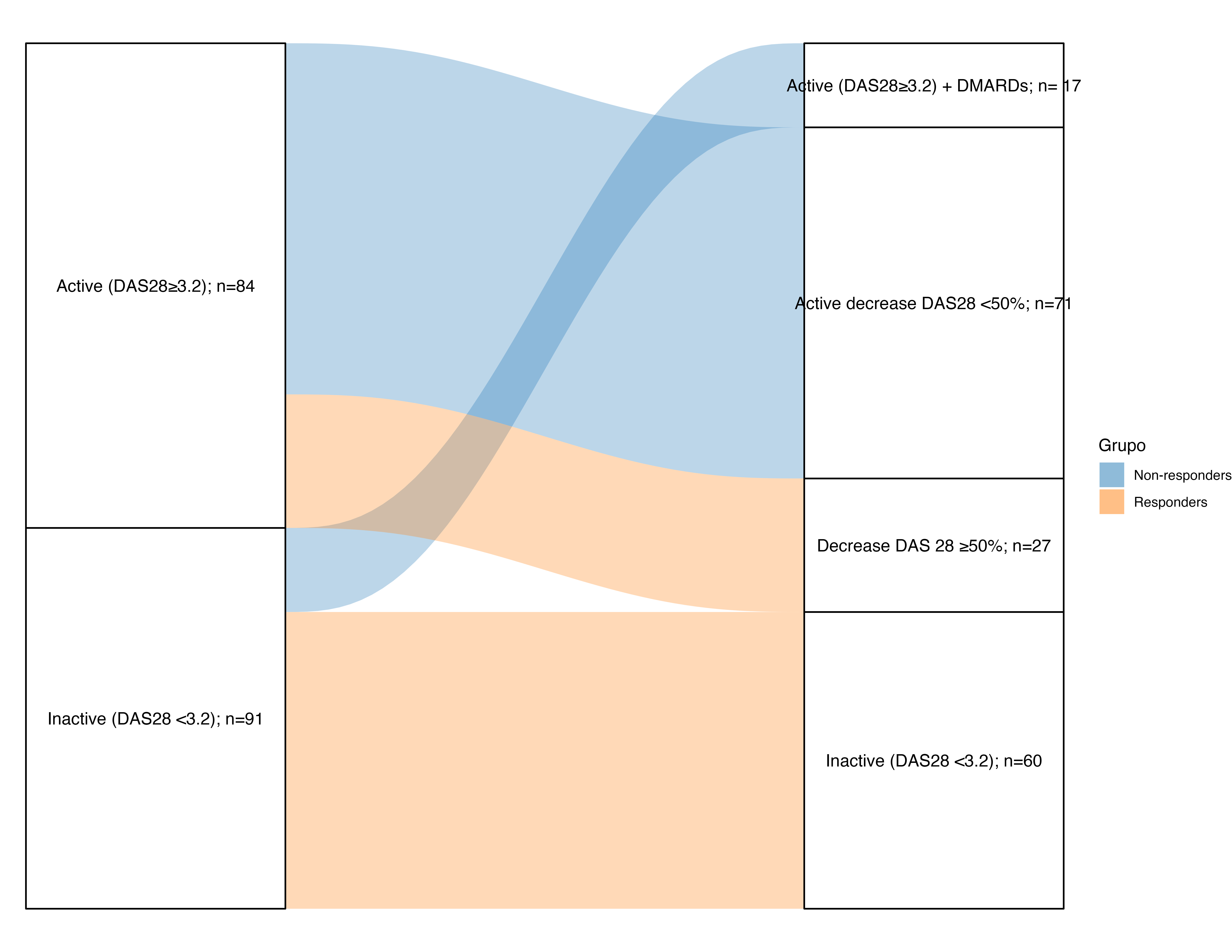

Supplement: Supplementary file 1 [file biomedicines-12-01406-s001.zip › biomedicines-3043876-supplementary/Supplementary_Figure1.jpeg]
